# Supplementary material for: Stacking Model‐Based Classifiers for Dealing With Multiple Sets of Noisy Labels
Source: Biom J. 2025 Mar 12;67(2):e70042. doi: 10.1002/bimj.70042 (PMC11898607; doi:10.1002/bimj.70042)
Supplement: Supplementary file 1 — Supporting Information [file BIMJ-67-e70042-s001.zip › density-based-ensemble-model-main/competitors_real_data/netlab-master/netlab-master/help/graddesc.htm]

Netlab Reference Manual graddesc


# graddesc

## Purpose

Gradient descent optimization.

## Description

`[x, options, flog, pointlog] = graddesc(f, x, options, gradf)` uses
batch gradient descent to find a local minimum of the function
`f(x)` whose gradient is given by `gradf(x)`. A log of the function values
after each cycle is (optionally) returned in `errlog`, and a log
of the points visited is (optionally) returned in `pointlog`.

Note that `x` is a row vector
and `f` returns a scalar value.
The point at which `f` has a local minimum
is returned as `x`. The function value at that point is returned
in `options(8)`.

`graddesc(f, x, options, gradf, p1, p2, ...)` allows
additional arguments to be passed to `f()` and `gradf()`.

The optional parameters have the following interpretations.

`options(1)` is set to 1 to display error values; also logs error
values in the return argument `errlog`, and the points visited
in the return argument `pointslog`. If `options(1)` is set to 0,
then only warning messages are displayed. If `options(1)` is -1,
then nothing is displayed.

`options(2)` is the absolute precision required for the value
of `x` at the solution. If the absolute difference between
the values of `x` between two successive steps is less than
`options(2)`, then this condition is satisfied.

`options(3)` is a measure of the precision required of the objective
function at the solution. If the absolute difference between the
objective function values between two successive steps is less than
`options(3)`, then this condition is satisfied.
Both this and the previous condition must be
satisfied for termination.

`options(7)` determines the line minimisation method used. If it
is set to 1 then a line minimiser is used (in the direction of the negative
gradient). If it is 0 (the default), then each parameter update
is a fixed multiple (the learning rate)
of the negative gradient added to a fixed multiple (the momentum) of
the previous parameter update.

`options(9)` should be set to 1 to check the user defined gradient
function `gradf` with `gradchek`. This is carried out at
the initial parameter vector `x`.

`options(10)` returns the total number of function evaluations (including
those in any line searches).

`options(11)` returns the total number of gradient evaluations.

`options(14)` is the maximum number of iterations; default 100.

`options(15)` is the precision in parameter space of the line search;
default `foptions(2)`.

`options(17)` is the momentum; default 0.5. It should be scaled by the
inverse of the number of data points.

`options(18)` is the learning rate; default 0.01. It should be
scaled by the inverse of the number of data points.

## Examples

An example of how this function can be used to train a neural network is:

```
options = zeros(1, 18);
options(17) = 0.1/size(x, 1);
net = netopt(net, options, x, t, 'graddesc');
```

Note how the learning rate is scaled by the number of data points.

## See Also

`conjgrad`, `linemin`, `olgd`, `minbrack`, `quasinew`, `scg`

---

**Pages:**
Index


---

Copyright (c) Ian T Nabney (1996-9)
